# Red Blood Cell Homeostasis and Altered Vesicle Formation in Patients With Paroxysmal Nocturnal Hemoglobinuria

**DOI:** 10.3389/fphys.2019.00578

**Published:** 2019-05-15

**Authors:** Joames K. Freitas Leal, Frank Preijers, Roland Brock, Merel Adjobo-Hermans, Giel Bosman

**Affiliations:** ^1^ Department of Biochemistry, Radboud University Medical Center, Nijmegen, Netherlands; ^2^ Laboratory for Hematology, Department of Laboratory Medicine, Radboud University Medical Center, Nijmegen, Netherlands

**Keywords:** red blood cells, paroxysmal nocturnal hemoglobinuria, aging, thrombosis, microvesicles

## Abstract

A subset of the red blood cells (RBCs) of patients with paroxysmal nocturnal hemoglobinuria (PNH) lacks GPI-anchored proteins. Some of these proteins, such as CD59, inhibit complement activation and protect against complement-mediated lysis. This pathology thus provides the possibility to explore the involvement of complement in red blood cell homeostasis and the role of GPI-anchored proteins in the generation of microvesicles (MVs) *in vivo*. Detailed analysis of morphology, volume, and density of red blood cells with various CD59 expression levels from patients with PNH did not provide indications for a major aberration of the red blood cell aging process in patients with PNH. However, our data indicate that the absence of GPI-anchored membrane proteins affects the composition of red blood cell-derived microvesicles, as well as the composition and concentration of platelet-derived vesicles. These data open the way toward a better understanding on the pathophysiological mechanism of PNH and thereby to the development of new treatment strategies.

## Introduction

Paroxysmal nocturnal hemoglobinuria (PNH) is a highly debilitating disease that is characterized by intravascular hemolysis, arterial, and venous thrombosis (Malato et al., 2012; Peacock-Young et al., 2018) and a variety of symptoms related to smooth muscle dystonia (DeZern and Brodsky, 2015). PNH is a rare disease with an incidence of 1–2 per 1,000,000 persons per year and is frequently associated with bone marrow failure such as aplastic anemia (Clemente et al., 2018). PNH is caused by clonal expansion of multipotent hematopoietic stem cells with somatic mutations in the *PIGA* gene. *PIGA* encodes for an enzyme that is critical in the synthesis of the first intermediate in the pathway of glycosylphosphatidylinositol (GPI) anchors. (Takeda et al., 1993; DeZern and Brodsky, 2015) As a consequence, the absence of *PIGA* activity results in hematopoietic cells that are deficient in GPI-anchored proteins. In RBCs, the absence of the GPI-anchored proteins decay-accelerating factor (DAF; CD55) and membrane inhibitor of reactive lysis (MIRL; CD59) that protect against complement-mediated lysis renders red blood cells (RBCs) highly vulnerable to intravascular hemolysis (Risitano and Rotoli, 2008; Brodsky, 2014). This results not only in anemia but also in the release of free hemoglobin and iron, which catalyzes the generation of reactive oxygen species and subsequent NO depletion and vasoconstriction (Kahn et al., 2013; Rapido, 2017). For untreated patients, thrombosis is the most common cause of death (Hill et al., 2013; Griffin and Munir, 2017).

The monoclonal antibody eculizumab is the most effective drug used in PNH (Brodsky, 2009). Eculizumab blocks the cleavage of C5 by the C5 convertase into C5b and thereby inhibits the formation of the terminal membrane attack complex (MAC) C5b-9 and consequent hemolysis of abnormal RBCs. This reduces RBC destruction and transfusion requirements (Carroll and Sim, 2011; Risitano, 2012; Bayly-Jones et al., 2017). Nevertheless, the opsonizing effects of activated complement factors such as C3d may induce RBC phagocytosis (Risitano et al., 2009; DeZern and Brodsky, 2015).

At present, the mechanism(s) responsible for clonal expansion during hematopoiesis and the variable clinical manifestations of the disease have only partially been elucidated (Hill et al., 2017), but increased removal of RBC may contribute to the pathophysiology of PNH (Risitano and Rotoli, 2008). RBC homeostasis is dependent on the generation of young and removal of aged RBCs. The latter process is initiated by binding of senescent cell-specific IgG, the appearance of molecules that may trigger pathological reactions, such as immunoreactive epitopes on damaged membrane proteins, and exposure of phosphatidylserine (PS) in the outer leaflet of the lipid bilayer, all leading to phagocytosis (Bosman et al., 2008; Dinkla et al., 2014; Klei et al., 2017). From biophysical, immunochemical, proteomic, and metabolomic studies, a molecular picture of the pathways involved in the normal aging and removal process of RBCs has emerged: oxidative damage-induced, high-affinity binding of hemoglobin to the cytoplasmic domain of band 3, activation of Ca^2+^-permeable channels, phosphorylation-controlled alterations in morphology and metabolism affecting ATP production and redox status, degradation of band 3 and/or aggregation of band 3 fragments, binding of IgG, and microvesicle (MV) generation (Ferru et al., 2011; Zolla and D’Alessandro, 2012; Bosman, 2016). Physiological anti-band 3 IgG has been reported to have a high affinity for dimeric C3b, thereby linking RBC phagocytosis to complement activation (Lutz and Bogdanova, 2013).

During physiological RBC aging, there is a small decrease in the content of GPI-anchored DAF and MIRL (Willekens et al., 2008), and in the content and activity of acetylcholinesterase (AChE), another GPI-anchored protein (Willekens et al., 2008; Freitas Leal et al., 2017). The latter observation suggests that the activities of DAF and/or MIRL might also decrease in healthy individuals and thereby contribute to complement-mediated opsonization and removal of old RBCs. AChE is increased in microvesicles, suggesting that changes in the distribution of GPI-anchored proteins in microdomains are associated with microvesicle (MV) generation (Salzer and Prohaska, 2001; Freitas Leal et al., 2017). As a consequence, the absence of GPI-anchored proteins may affect the microvesiculation process. Indeed, some data indicate that microvesiculation of RBCs and platelets may be impaired in PNH patients (Whitlow et al., 1993). Also, it has been shown that activated complement induces the massive formation of vesicles with a strong pro-coagulant activity (Ninomiya et al., 1999). Thus, the absence of GPI-anchored proteins may have a pronounced effect on RBC morphology, function, and survival (Whitlow et al., 1993). In addition, exposure of the pro-coagulant and removal signal PS, which is in general associated with abnormal membrane organization and vesiculation in damaged or stressed, but not in aged RBCs (Bosman et al., 2008), has been reported to be increased in RBCs of PNH patients (Sato et al., 2010).

Here, we have selected a number of aging-associated parameters from this current knowledge of the molecular mechanisms involved in physiological RBC homeostasis (Bosman et al., 2008, 2012; Lutz and Bogdanova, 2013; Bosman, 2016; Freitas Leal et al., 2018) that might be relevant for the pathophysiology of PNH, in order to explore the effect of the absence of GPI-linked proteins on RBC structure, function, aging, and removal *in vivo*. Our data, obtained from PNH patients with various clone sizes and following various treatment regimes, indicate no significant effects of the absence of GPI-linked proteins on RBC turnover but emphasize the heuristic value of more, detailed studies on the origin, composition, and activity of RBC-derived and platelet-derived microvesicles.

## Materials and Methods

### Red Blood Cell Sampling

Blood was collected by venipuncture from healthy volunteers and 15 patients after obtaining written informed consent, and using EDTA as anticoagulant, following the guidelines of the local medical ethical committee (CMO regio Arnhem Nijmegen) and in accordance with the Declaration of Helsinki. Leukocytes and platelets were removed as described before using Ficoll-Paque (Freitas Leal et al., 2017). The time between blood collection, fractionation, and analysis was identical for all samples.

### Red Blood Cell Fractionation and Microscopic Analysis

RBCs were fractionated according to cell density using discontinuous Percoll gradients ranging from 40% Percoll (1.060 g/ml) to 80% Percoll (1.096 g/ml) as described before (Willekens et al., 2008; Freitas Leal et al., 2017). The various RBC fractions were isolated and washed three times with Ringer’s solution (Freitas Leal et al., 2017) by repeated centrifugation for 5 min at 400 *g* before analysis. RBC morphology was analyzed using a TCS SP5 confocal laser scanning microscope (Leica Microsystems, Mannheim, Germany) as described before (Cluitmans et al., 2015).

### Isolation and Characterization of Microvesicles From Plasma

Microvesicles (MVs) were isolated from the platelet-rich plasma (PRP) obtained after differential centrifugation as described before (Dinkla et al., 2012, 2013, 2016).

### Flow Cytometry Analysis

Classification of the RBCs according to PNH type was performed by flow cytometry using FITC-labeled CD235a (clone KC16, 1:100, Beckman Coulter, Fullerton, CA, USA) and PE-labeled CD59 (clone MEM43, 1:400, IQ products, Groningen, the Netherlands) as described before (Sutherland et al., 2015). PNH RBCs were classified based on CD59 content in type III (complete GPI-deficiency), type II (partial GPI-deficiency), and type I (normal expression) cells (Sutherland et al., 2015). APC-labeled CD71 (clone CY1G4, 1:200, Biolegend, San Diego, California, USA) was combined with PE-labeled CD59 to evaluate the percentage of reticulocytes per PNH type. FITC-labeled anti-C3c (1:200, Abcam, Cambridge, UK) and APC-labeled anti-C3d (1 μg/million cells, Assay Pro, St. Louis, Missouri, USA) were combined with PE-labeled CD59 to evaluate the degree of opsonization per PNH type. Staining of band 3 with eosin-5′ maleimide (EMA, Thermo Fisher Scientific, Landsmeer, the Netherlands) was performed by incubating 1 million RBCs with 25 μl of EMA (0.5 mg/ml in Ringer’s solution) in the dark at RT for 15 min. (Cobb and Beth, 1990; Crisp et al., 2011). After staining, RBCs were washed three times with Ringer’s solution and analyzed by flow cytometry [FACSCalibur instrument (BD Biosciences, Franklin Lakes NJ, USA)] using CELLQuest software (BD Biosciences). Data were analyzed with FlowJo cell analysis software v.10 (FlowJo, LLC, Ashland, OR) using 200,000 events. Microvesicle analysis was performed using mixtures of PE-labeled CD59 (1:400), FITC-labeled CD235a (1:100), and PE/Cy5-labeled CD41 (1:10) by flow cytometry as previously described (Dinkla et al., 2012, 2013). Sulfate latex microspheres (0.9 μm, Invitrogen, Carlsbad CA, USA) and washed Flow-Count calibration beads (Beckman Coulter, Brea CA, USA) were used for quantification (Dinkla et al., 2012). Microvesicles were classified based on CD59 positivity in CD59-negative (complete GPI-deficiency), low CD59 (partial GPI-deficiency), and wild type (normal expression).

### Comparisons and Statistical Analyses

The exclusion criteria for the PNH patients were other hematological comorbidities besides aplastic anemia and having received a red blood cell transfusion within a period of 3 months before analysis. For most analyses, we compared PNH patients with control donors and PNH patients being treated with eculizumab with patients without eculizumab. Differences between groups were determined using a two-way ANOVA test. Non-parametric *t*-tests or one-way ANOVA tests were used to analyze differences between control and PNH samples. Wilcoxon matched pair tests were used to analyze differences between the various RBC fractions inside the groups, and the Fisher LSD test was used to compare controls and patient samples. Two-sided *p*’sless than 0.05 were used to determine statistical significance. Relations between the various parameters were estimated using the Pearson correlation coefficient.

## Results

### RBC Morphology and Phenotype

**Figure 1:**
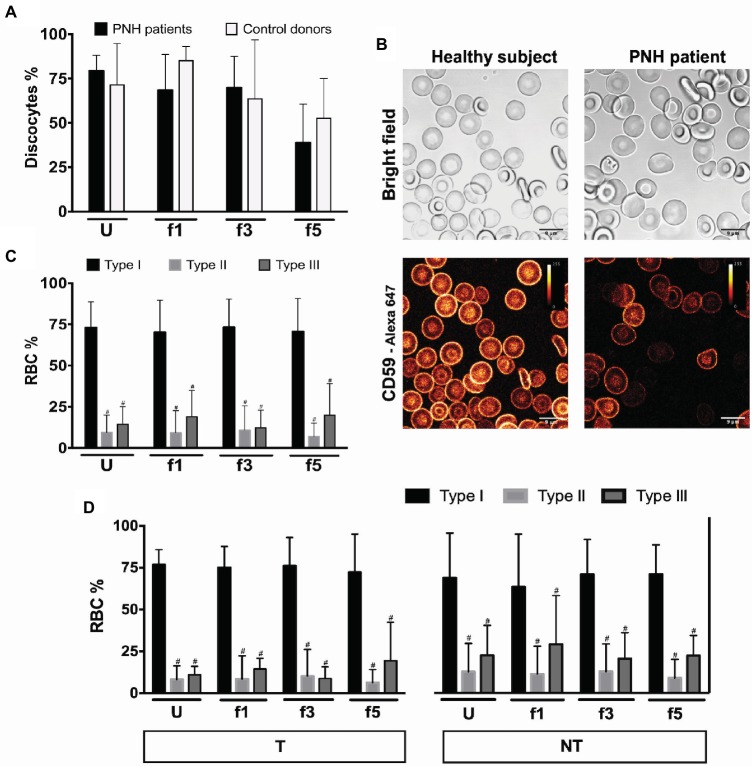
RBCs morphology and phenotype of PNH patients. **(A)** Percentage of discocytes per Percoll fraction in PNH patients (*N* = 5) and healthy control donors (*N* = 5); **(B)** bright field and fluorescence images of anti-CD59-Alexa 647 stained RBCs from a healthy subject and a PNH patient, showing CD59 density; **(C)** RBCs of PNH patients (*N* = 9) were separated according to density and analyzed by flow cytometry regarding their CD59 content (type I, II, and III); **(D)** RBCs of PNH patients being treated with eculizumab (T; *N* = 5) and non-treated PNH patients (NT; *N* = 4) separated according to density and analyzed by flow cytometry according to their CD59 content (type I, II, and III). ^#^Significantly different from type I in the same Percoll fraction (*p* < 0.05). U, unseparated; f1, f3, f5, fractions of increasing density isolated by Percoll density separation (Materials and Methods).

During aging *in vivo* and *in vitro,* RBCs undergo a series of morphological changes that result in the appearance of deformed, mostly spherocytic cells. Semi-quantitative analysis of these changes has been shown to be informative on RBC hemostasis and on the relationship between morphology, deformability, and survival (Cluitmans et al., 2015). Microscopic analysis of RBCs from patients with PNH showed a tendency to a decrease in the numbers of cells with the regular discocyte form and a concomitant increase in the numbers of echinocyte-like and otherwise misshapen cells, especially in the densest cell fractions (Figure 1A). The majority of the patients’ RBCs were type I according to CD59 expression levels (Figure 1B), and we found no differences in the percentages of type II and type III cells between the various Percoll layers (Figure 1C). Treatment with eculizumab did not result in significant differences in CD59-deficient cells (Figure 1D).

### Membrane/Band 3 Content (Eosine 5′-Maleimide)

**Figure 2:**
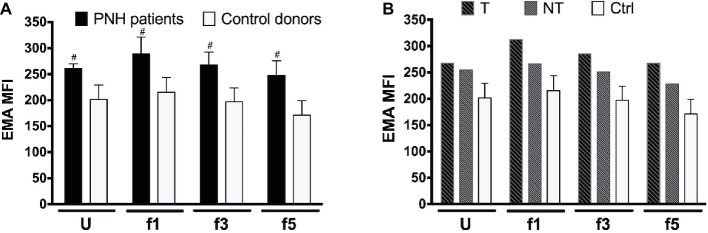
Eosin 5′-maleimide Mean Fluorescence Intensity (MFI) of RBC fractions. **(A)** RBCs of PNH patients (*N* = 2) and of control healthy donors (*N* = 7) of various Percoll fractions were stained with eosin 5′-maleimide (EMA). The degree of staining is expressed as the mean fluorescence intensity (MFI). **(B)** EMA MFI of RBCs of a PNH patient being treated with (T) and without (NT) eculizumab, separated according to density. Ctrl, healthy donors (*N* = 7). The samples were analyzed as described before (see Materials and Methods). ^#^Significantly different from control (*p* < 0.05). U, unseparated; f1, f3, f5, fractions of increasing density isolated by Percoll density separation (Materials and Methods).

RBC aging is accompanied by changes in membrane organization that are associated with the appearance of removal signals and with the loss of cell membrane. Especially, changes in the integral membrane protein band 3 play a pivotal role in the generation of senescence-specific antigens, in the interaction between lipid bilayer and cytoskeleton, and in the generation of microvesicles (Willekens et al., 2008; Bosman et al., 2012; Lutz and Bogdanova, 2013; Freitas Leal et al., 2018). The amount of binding of the band 3 probe eosine 5′-maleimide (EMA) is mostly a sensitive marker of band 3 content, but also of Rh, Rh glycoprotein, and CD47, and/or of the loss of membrane (Cobb and Beth, 1990; Huisjes et al., 2018). Flow cytometric analysis of the binding of EMA showed a higher EMA signal in all RBC fractions from two different PNH patients tested, independent of cell density and treatment (Figure 2). There was no significant difference in the density-associated decrease between control donors or any of the PNH patients. Also, there was no statistically significant correlation between EMA fluorescence and the RBC size (forward scatter) in the RBC fractions of controls and PNH patients taken together (*r* = 0.31, *p* = 0.18, *N* = 20).

### Complement Deposition (C3c and C3d)

**Figure 3:**
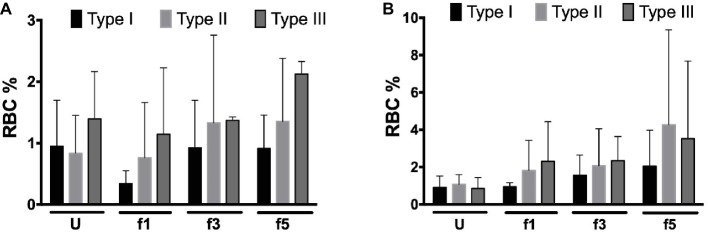
Complement deposition on density-separated RBCs. **(A)** Percentage of C3c-positive RBCs in the PNH RBC population divided per CD59 content (type I, II, and III) per density (Percoll fraction; *N* = 2); **(B)** percentage of C3d-positive RBCs in the PNH RBC population according to CD59 content (type I, II, and III) per Percoll fraction (type I, II, and III; *N* = 3). The samples were analyzed as described before (see Materials and Methods). U, unseparated; f1, f3, f5, fractions of increasing density isolated by Percoll density separation (Materials and Methods).

Activation of complement may lead to deposition of complement fragments on RBC through the CR1 receptor, and the presence of C3b fragments induces phagocytosis of eculizumab-treated, CD59-negative RBCs *in vitro* (Lin et al., 2015). We therefore also probed for the presence of C3c and C3d in density-separated RBCs. For both proteins, we observed a tendency to an increase in the percentage of positive cells with cell density (Figure 3). Thus, the content of RBC-bound C3c as well as C3d may increase with cell age, also on type I RBCs with a normal content of CD59 (Figure 3). These findings are in agreement with previous indications for the involvement of complement in phagocytosis *in vitro* (Lutz, 2004; Arese et al., 2005). We found no significant correlations between these parameters and treatment with eculizumab (data not shown).

### Reticulocytes

**Figure 4:**
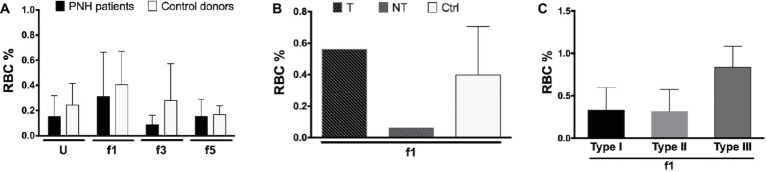
Reticulocytes in patients with PNH. **(A)** Percentage of CD71-expressing RBCs from the blood of PNH patients (*N* = 2) and healthy control donors RBCs (*N* = 4) of various Percoll fractions after staining with APC-labeled CD71; **(B)** percentage of APC-CD71-positive RBCs of a PNH patient being treated with eculizumab (T), a non-treated PNH patient (NT), and healthy control donors in the reticulocyte-enriched Percoll fraction 1 (Ctrl; *N* = 4); **(C)** percentage of APC-CD71-positive RBCs in the PNH RBC population per CD59 content (type I, II, and III) in fraction 1 (*N* = 2). The samples were analyzed as described before (see Materials and Methods). U, unseparated; f1, f3, f5, fractions of increasing density isolated by Percoll density separation (Materials and Methods).

Aberrant RBC structure resulting in a decreased mean life and leading to anemia is, in many cases, compensated by increased erythropoiesis, as indicated by changes in the size of the reticulocyte fraction. The hematological data show a large variability in the size of the reticulocyte fractions of our patients, without any significant correlation with other patient variables, although most eculizumab-treated patients had higher reticulocyte numbers than the patients without eculizumab (Supplementary Table 1). Flow cytometric analysis of the RBCs of a few PNH patients showed similar data, also without significant differences between donors or RBC fractions (Figures 4A,B). In general, most reticulocytes were found in the lightest density fractions upon Percoll separation, i.e., fraction 1 (Figure 4A), as shown before for healthy individuals (Willekens et al., 2008). The fraction of type III, CD59-lacking reticulocytes was considerably higher than the other types (Figure 4C), which may reflect a disturbed differentiation and/or maturation process in the absence of GPI-linked proteins (Sato et al., 2010).

### Microvesicles

**Figure 5:**
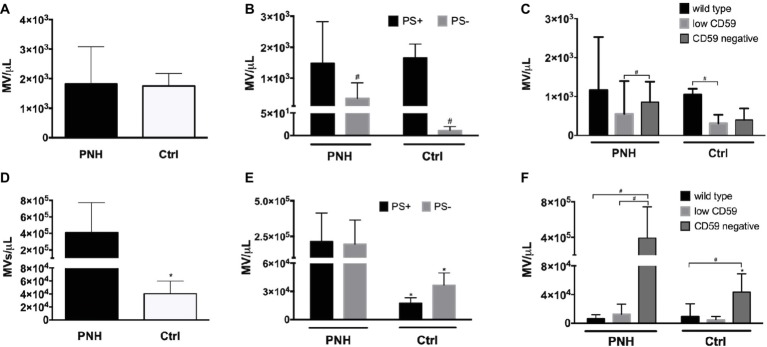
Microvesicle numbers and composition in the blood of patients with PNH. **(A)** Concentration per microliter (MV/μl) of RBC-derived, CD235a-positive microvesicles in the blood of PNH patients (*N* = 9) and control healthy donors (N = 6); **(B)** concentration of RBC-derived microvesicles in the blood of PNH patients (*N* = 9) and control healthy donors (*N* = 3), distinguished according to their reactivity to Annexin V (phosphatidylserine-positive (PS+) or negative (PS−); **(C)** RBC-derived microvesicles were categorized into wild type, CD59-low and CD59-negative PNH, *N* = 9; Ctrl, *N* = 3), as described for RBCs (Materials and Methods); **(D)** concentration of CD41-positive, platelet-derived microvesicles in the blood of PNH patients (*N* = 9) and control healthy donors (*N* = 6); **(E)** concentration of platelet-derived microvesicles according their reactivity to Annexin V (PS+ or PS−; PNH, *N* = 9; Ctrl, *N* = 3); **(F)** platelet-derived microvesicles were categorized into wild type, CD59-low and CD59-negative as described for RBCs and quantified and analyzed by flow cytometry as described before (PNH, *N* = 9; Ctrl, *N* = 3). ^#^Significantly different from the other parameter (*p* < 0.05); ^*^Significantly different from the patients’ samples (*p* < 0.05).

Microvesicle generation is an integral part of the physiological RBC aging process, and changes in microvesicle concentration as well as composition occur in patients with disturbed RBC homeostasis (Freitas Leal et al., 2018). We found no significant differences in the concentrations of RBC-derived microvesicles between PNH patients and controls (Figure 5A). However, the concentration of PS-negative microvesicles in the plasma of PNH patients was higher than in the plasma of control donors (Figure 5B). The concentration of CD59-high RBC-derived microvesicles was higher than that of the other types in the plasma of control donors but not in the plasma of PNH patients (Figure 5C). Platelet-derived microvesicle concentrations were much higher in the plasma of PNH patients than in controls (Figure 5D), both the PS-positive and the PS-negative microvesicles (Figure 5E). Remarkably, almost all platelet-derived microvesicles were devoid of CD59, including those from the plasma of control donors (Figure 5F). We observed no statistically significant correlations between the numbers of RBC-derived and platelet-derived vesicles (*r* = −0.40, *p* = 0.28, *N* = 9).

## Discussion

### RBC Aging and Generation of Microvesicles

Red blood cells of PNH patients lack the key GPI-anchored membrane proteins that protect against activated complement. We postulated that this change in membrane composition has a more wide-spread effect on membrane organization and thereby on various aspects of RBC homeostasis. The most obvious aspects derive from the role of complement in removal of senescent RBCs and the involvement of GPI-linked proteins in microdomain-associated generation of microvesicles (Lutz, 2004; Lutz and Bogdanova, 2013; de Back et al., 2014; Saha et al., 2016; Pollet et al., 2018). In this exploratory study, we did not find significant indications for a pronounced alteration of RBC homeostasis in patients with PNH, as based on cell volume, cell density, and morphology or on clinical hematology parameters, including LDH values (Supplementary Table 1). Thus, in most of our patients, the lack of GPI-anchored proteins does not seem to cause a major disturbance of the physiological RBC aging mechanisms.

Nevertheless, there were clear differences related to membrane composition and microvesicle formation. The EMA measurements showed significant differences between the RBCs of PNH patients and of control donors (Figure 2). The tendency to a density-associated decrease in EMA staining might be due to loss of band 3 and/or membrane with aging by vesiculation, both in RBCs from control donors and from PNH patients. This has been postulated before for physiological aging *in vivo* (Willekens et al., 2008). However, the absence of a statistically significant correlation between EMA fluorescence and the RBC size, based on the cytometer parameter forward scatter, suggests that in the RBCs from PNH patients, the band 3 protein content is not a direct function of cell size. EMA staining is affected by changes in band 3 conformation and membrane organization as well (e.g., Cobb and Beth, 1990; Huisjes et al., 2018). Combined with the considerable fractions of PS-negative and CD59-lacking microvesicles in the blood of PNH patients (Figure 5), these data indicate that the organization of the RBC membrane, as well as the mechanism of microvesicle generation, are altered by the absence of GPI-linked proteins. This may be a direct effect, but also the consequence of the deposition of C3b. The latter not only affects lateral mobility of CD59 and band 3 molecules but also membrane viscosity and deformability (Karnchanaphanurach et al., 2009; Glodek et al., 2010). Our *in vivo* data support the involvement of GPI-linked proteins in microvesicle formation during RBC aging *in vitro* (Salzer et al., 2008; Freitas Leal et al., 2017). The differences in mechanisms leading to the generation of microvesicles with and without PS at their outside remain to be established, as well as the effect on biological activity. Since PS exposure contributes to recognition and removal of microvesicles by macrophages (Willekens et al., 2005), its absence may not only affect their pro-coagulant activity but also their lifespan. Fusion between microvesicles and RBCs may underlie the reported transfer between CD55 and CD59 from normal RBCs to RBCs without these proteins (Sloand et al., 2004). Thus, microvesicles generated by PNH RBCs may also fuse with normal RBCs, thereby affecting their membrane organization as well. Furthermore, increased levels of RBC-derived microvesicles may affect NO bioavailability (Said et al., 2018) and induce activation of endothelial cells and tissue factor expression (Collier et al., 2013), thereby contributing to the wide-spread thrombosis in patients with PNH.

### Platelet Microvesicles and Thrombosis

Platelets without CD59 have been described to catalyze the rate of prothrombin conversion upon treatment with complement C5b-9 *in vitro*, and this was associated with an increase in microvesicle formation (Wiedmer et al., 1993). RBC-derived and platelet-derived, phosphatidylserine-positive microvesicles have been reported to be increased approximately two-fold in the blood of PNH patients (Hugel et al., 1999). We found equal concentrations of RBC-derived microvesicles in the plasma of PNH patients and healthy donors, but much larger RBC-derived, phosphatidylserine-negative microvesicle concentrations in the blood of PNH patients (Figure 5B), and larger concentrations of platelet-derived vesicles (Figure 5D). In the plasma of eculizumab-treated PNH patients, the numbers of RBC-derived vesicles were lower than in patients who had not been treated with eculizumab (Supplementary Figure S1). The absence of a statistically significant correlation between the concentrations of RBC-derived and platelet-derived microvesicles indicates that the absence of GPI-linked proteins affects microvesicle generation from RBCs and platelets through different mechanisms. Although in control donors, most platelets are CD59-positive (Jin et al., 1997), almost all platelet-derived microvesicles were CD59-negative (Figure 5). There were approximately equal concentrations of platelet-derived vesicles with and without PS at their surface (Figure 5E). These data strongly suggest that the absence of GPI-linked proteins does not only have a pronounced stimulatory effect on the generation of microvesicles but also on their composition. The latter may be related to the presence of tissue factor and is likely to affect their function (Devalet et al., 2014). Our recent finding that platelet-derived microvesicles can prevent differentiation of regulatory T-cells through P-selectin (Dinkla et al., 2016) emphasizes their pivotal role in the pathophysiology of many diseases that may include PNH (Devalet et al., 2014). Although the name suggests otherwise, most platelet-derived microvesicles originate not from platelets, but from megakaryocytes in the bone marrow (Flaumenhaft et al., 2009; Rank et al., 2010). It is not known how the absence of GPI-linked proteins affects megakaryocyte biology and/or platelet activation. These data support the importance of an extensive characterization of origin, composition, and biological activity of CD41-positive microvesicles. Such studies may help in establishing an urgently needed, robust marker of platelet activation.

## Conclusion

The heterogeneity of the patient population and the concomitant small numbers available for statistical comparisons of all parameters preclude a robust answer on the question whether RBC aging is altered in patients with PNH. However, the combined results of the selected aging-associated parameters (Bosman et al., 2008, 2012; Lutz and Bogdanova, 2013) do not reveal a major aberration of the physiological RBC aging process in patients with PNH. Remarkably, formation of microvesicles by RBCs is altered in patients with PNH. This is likely due to PNH-related differences in membrane organization that is associated with the absence of GPI-linked proteins. The conspicuous lack of phosphatidylserine exposure on many RBC-derived microvesicles in PNH patients may affect their time in the circulation as well as their contribution to hemostasis and thrombosis. In platelets, PNH-related processes seem not only to induce the appearance of large numbers of phosphatidylserine-negative microvesicles but also to cause excessive formation of microvesicles. Future investigations leading to a better understanding of the mechanisms underlying vesiculation, effect of vesiculation on RBC function and survival, and effect of the various microvesicles on thrombosis in patients with PNH may be instrumental in developing new treatment strategies (Kulasekararaj et al., 2019).

## Data Availability

The raw data supporting the conclusions of this manuscript will be made available by the authors, without undue reservation, to any qualified researcher.

## Ethics Statement

This study was carried out in accordance with the recommendations of the medical ethical committee “CMO-Regio Arnhem Nijmegen”; with written informed consent from all subjects. All subjects gave written informed consent in accordance with the Declaration of Helsinki. The protocol was approved by the CMO-Regio Arnhem Nijmegen.

## Author Contributions

JF performed all measurements, the analyses, and wrote the first version of the manuscript. FP provided the samples, some protocols, and assisted in writing the manuscript. RB, MA-H, and GB contributed to the setup of the study, the interpretation of the data, and the writing of the manuscript.

### Conflict of Interest Statement

The authors declare that the research was conducted in the absence of any commercial or financial relationships that could be construed as a potential conflict of interest.

## Supplementary Material

SUPPLEMENTARY FIGURE S1 Microvesicle numbers and composition in the blood of patients with PNH CD59 level and treatment. **(A)** Concentration (MV/μl) of CD235a-positive, RBC-derived microvesicles (MVs) in the blood of PNH patients who did not receive treatment (NT; *N* = 3), PNH patients who were treated with eculizumab (T; *N* = 6), and control healthy donors (*N* = 6); **(B)** concentration of RBC-derived microvesicles in the blood of PNH patients who did not receive treatment (NT; *N* = 3), PNH patients who were being treated with eculizumab (T; *N* = 6) and control donors (*N* = 6) according their reactivity to Annexin V (phosphatidylserine positive (PS+) or negative (PS−); **(C)** RBC-derived microvesicles in the blood of PNH patients who did not receive treatment (NT; *N* = 3), PNH patients who were being treated with eculizumab (T; *N* = 6) categorized in wild type, CD59-low and CD59-negative as described for RBCs (Materials and Methods); **(D)** concentration of platelet-derived microvesicles (CD41-positive) from the blood of PNH patients who did not receive treatment with eculizumab (NT; *N* = 3), PNH patients who were being treated with eculizumab (T; *N* = 6) and control healthy donors (*N* = 6); **(E)** concentration of platelet-derived microvesicles in the blood of PNH patients who did not receive treatment with eculizumab (NT; *N* = 3), PNH patients who were being treated with eculizumab (T; *N* = 6) and control healthy donors (*N* = 6), according their reactivity to Annexin V (PS+ or PS−); **(F)** platelet-derived microvesicles from the blood of PNH patients who did not receive treatment with eculizumab (NT; *N* = 3), PNH patients who were being treated with eculizumab (T; *N* = 6) were categorized in wild type, low CD59, and CD59-negative as described for RBCs and quantified and analyzed by flow cytometry as described before (see Materials and Methods). ^#^Significantly different from the other parameter in the same group (*p* < 0.05). ^*^Significantly different between groups (*p* < 0.05).

SUPPLEMENTARY TABLE 1 Clinical data of PNH patients. AA, PNH patient with aplastic anemia; *N*, PNH patient without hematological comorbidities; T, in treatment; NT, not in treatment; RBC, red blood cells (×10^12^/L); RBC CS (II/III), red blood cell clone size (type II and III); Hb, hemoglobin (g/dl); Ht, hematocrit (%); MCV, mean corpuscular volume (fl); MCH, mean corpuscular hemoglobin (pg); MCHC, mean corpuscular hemoglobin concentration (g/dl); RDW, red blood cell distribution width (%); Retic, reticulocytes (promille); RBC Tr, red blood cell transfusion in the last 3 months; Leuk, Leukocytes (×10^9^/L); Gran CS., granulocytes clone size; Plt, platelet (×10^9^/L); LDH, lactate dehydrogenase (U/L); −, not available. Reference values for healthy adults: RBC: for men, 4.7–6.1 × 10^12^/L and for women, 4.2–5.4 × 10^12^/L; Hb: for men, 8.5–11 mmol/L and for women, 7.5–10 mmol/dl; Ht: for men, 0.4–0.54 and for women, 0.36–0.46; MCV: 80–96 fl; MCH: 1.7–2.1 fmol; MCHC: 19.3–22.5 mmol/L; RDW: 11.5–14.5%; Retic: 8–26 promille; Leuk: 4.5–11 × 10^9^/L; Plt: 150–400 × 10^9^/L; LDH: 135–225 U/L.

## References

[ref1] Arese P. Turrini F. Schwarzer E. (2005). Band 3/complement-mediated recognition and removal of normally senescent and pathological human erythrocytes. Cell. Physiol. Biochem. 16, 133–146. 10.1159/000089839, PMID: 16301814

[ref2] Bayly-Jones C. Bubeck D. Dunstone M. A. (2017). The mystery behind membrane insertion: a review of the complement membrane attack complex. Philos. Trans. R. Soc. Lond. Ser. B Biol. Sci. 372:20160221. 10.1098/rstb.2016.0221 28630159 PMC5483522

[ref3] Bosman G. (2016). The proteome of the red blood cell: an auspicious source of new insights into membrane-centered regulation of homeostasis. Proteome 4:35. 10.3390/proteomes4040035, PMID: 28248245 PMC5260968

[ref4] Bosman G. J. C. G. M. Lasonder E. Groenen-Döpp Y. A. M. Willekens F. L. A. Werre J. M. (2012). The proteome of erythrocyte-derived microparticles from plasma: new clues for erythrocyte aging and vesiculation. J. Proteome 76, 203–210. 10.1016/j.jprot.2012.05.031 22669077

[ref5] Bosman G. J. C. G. M. Werre J. M. Willekens F. L. A. Novotný V. M. J. (2008). Erythrocyte ageing *in vivo* and *in vitro*: structural aspects and implications for transfusion. Transfus. Med. 18, 335–347. 10.1111/j.1365-3148.2008.00892.x, PMID: 19140816

[ref6] Brodsky R. A. (2009). How I treat paroxysmal nocturnal hemoglobinuria. Blood 113, 6522–6527. 10.1182/blood-2009-03-195966, PMID: 19372253 PMC2710914

[ref7] Brodsky R. A. (2014). Paroxysmal nocturnal hemoglobinuria. Blood 124, 2804–2811. 10.1182/blood-2014-02-522128, PMID: 25237200 PMC4215311

[ref8] Carroll M. V. Sim R. B. (2011). Complement in health and disease. Adv. Drug Deliv. Rev. 63, 965–975. 10.1016/j.addr.2011.06.005, PMID: 21704094

[ref9] Clemente M. J. Przychodzen B. Hirsch C. M. Nagata Y. Bat T. Wlodarski M. W.. (2018). Clonal PIGA mosaicism and dynamics in paroxysmal nocturnal hemoglobinuria. Leukemia 32, 2507–2511. 10.1038/s41375-018-0138-5, PMID: 29749402 PMC8694093

[ref10] Cluitmans J. C. A. Tomelleri C. Yapici Z. Dinkla S. Bovee-Geurts P. Chokkalingam V.. (2015). Abnormal red cell structure and function in neuroacanthocytosis. PLoS One 10:e0125580. 10.1371/journal.pone.0125580, PMID: 25933379 PMC4416783

[ref11] Cobb C. E. Beth A. H. (1990). Identification of the Eosinyl-5-maleimide reaction site on the human erythrocyte anion-exchange protein: overlap with the reaction sites of other chemical probes. Biochemistry 29, 8283–8290. 10.1021/bi00488a012, PMID: 1701324

[ref12] Collier M. E. W. Mah P. M. Xiao Y. Maraveyas A. Ettelaie C. (2013). Microparticle-associated tissue factor is recycled by endothelial cells resulting in enhanced surface tissue factor activity. Thromb. Haemost. 110, 966–976. 10.1160/TH13-01-0055, PMID: 23945646

[ref13] Crisp R. L. Solari L. Vota D. García E. Miguez G. Chamorro M. E. (2011). A prospective study to assess the predictive value for hereditary spherocytosis using five laboratory tests (cryohemolysis test, eosin-5′-maleimide flow cytometry, osmotic fragility test, autohemolysis test, and SDS-PAGE) on 50 hereditary spherocytosis fa. Ann. Hematol. 90, 625–634. 10.1007/s00277-010-1112-0 21080168

[ref14] de Back D. Z. Kostova E. B. van Kraaij M. van den Berg T. K. van Bruggen R. (2014). Of macrophages and red blood cells; a complex love story. Front. Physiol. 5:9. 10.3389/fphys.2014.00009, PMID: 24523696 PMC3906564

[ref15] Devalet B. Mullier F. Chatelain B. Dogne J.-M. Chatelain C. (2014). The central role of extracellular vesicles in the mechanisms of thrombosis in paroxysmal nocturnal haemoglobinuria: a review. J. Extracell. Vesicles 3, 1–8. 10.3402/jev.v3.23304 PMC3965713 24672668

[ref16] DeZern A. E. Brodsky R. A. (2015). Paroxysmal nocturnal hemoglobinuria. A complement-mediated hemolytic anemia. Hematol. Oncol. Clin. North Am. 29, 479–494. 10.1016/j.hoc.2015.01.005 26043387 PMC4695989

[ref17] Dinkla S. Brock R. Joosten I. Bosman G. J. C. G. M. (2013). Gateway to understanding microparticles: standardized isolation and identification of plasma membrane-derived vesicles. Nanomedicine 8, 1657–1668. 10.2217/nnm.13.149, PMID: 24074388

[ref18] Dinkla S. Peppelman M. Der Raadt J. Atsma F. Novotńy V. M. J. Van Kraaij M. G. J.. (2014). Phosphatidylserine exposure on stored red blood cells as a parameter for donor-dependent variation in product quality. Blood Transfus. 12, 204–209. 10.2450/2013.0106-13, PMID: 24120596 PMC4039702

[ref19] Dinkla S. Van Cranenbroek B. Van Der Heijden W. A. He X. Wallbrecher R. Dumitriu I. E. (2016). Platelet microparticles inhibit IL-17 production by regulatory T cells through P-selectin. Blood 127, 1976–1986. 10.1182/blood-2015-04-640300 26903549

[ref20] Dinkla S. Wessels K. Verdurmen W. P. R. Tomelleri C. Cluitmans J. C. A. Fransen J.. (2012). Functional consequences of sphingomyelinase-induced changes in erythrocyte membrane structure. Cell Death Dis. 3:e410. 10.1038/cddis.2012.143, PMID: 23076218 PMC3481131

[ref21] Ferru E. Giger K. Pantaleo A. Campanella E. Grey J. Ritchie K.. (2011). Regulation of membrane-cytoskeletal interactions by tyrosine phosphorylation of erythrocyte band 3. Blood 117, 5998–6006. 10.1182/blood-2010-11-317024, PMID: 21474668 PMC3112043

[ref22] Flaumenhaft R. Dilks J. R. Richardson J. Alden E. Patel-Hett S. R. Battinelli E. (2009). Megakaryocyte-derived microparticles: direct visualization and distinction from platelet-derived microparticles. Blood 113, 1112–1121. 10.1182/blood-2008-06-163832 18802008 PMC2635076

[ref23] Freitas Leal J. K. Adjobo-Hermans M. J. W. Bosman G. J. C. G. M. (2018). Red blood cell homeostasis: mechanisms and effects of microvesicle generation in health and disease. Front. Physiol. 9:703. 10.3389/fphys.2018.00703, PMID: 29937736 PMC6002509

[ref24] Freitas Leal J. K. Adjobo-Hermans M. J. W. Brock R. Bosman G. J. C. G. M. (2017). Acetylcholinesterase provides new insights into red blood cell ageing *in vivo* and *in vitro*. Blood Transfus. 15, 232–238. 10.2450/2017.0370-16, PMID: 28518050 PMC5448829

[ref25] Glodek A. M. Mirchev R. Golan D. E. Khoory J. A. Burns J. M. Shevkoplyas S. S.. (2010). Ligation of complement receptor 1 increases erythrocyte membrane deformability. Blood 116, 6063–6071. 10.1182/blood-2010-04-273904, PMID: 20861458 PMC3031392

[ref26] Griffin M. Munir T. (2017). Management of thrombosis in paroxysmal nocturnal hemoglobinuria: a clinician’s guide. Ther. Adv. Hematol. 8, 119–126. 10.1177/2040620716681748 28246555 PMC5305005

[ref27] Hill A. DeZern A. E. Kinoshita T. Brodsky R. A. (2017). Paroxysmal nocturnal haemoglobinuria. Nat. Rev. Dis. Primers. 3:17028. 10.1038/nrdp.2017.28, PMID: 28516949 PMC7879566

[ref28] Hill A. Kelly R. J. Hillmen P. (2013). Thrombosis in paroxysmal nocturnal hemoglobinuria. Blood 121, 4985–4996. 10.1182/blood-2012-09-311381, PMID: 23610373

[ref29] Hugel B. Socié G. Vu T. Toti F. Gluckman E. Freyssinet J. M.. (1999). Elevated levels of circulating procoagulant microparticles in patients with paroxysmal nocturnal hemoglobinuria and aplastic anemia. Blood 93, 3451–3456. PMID: 10233897

[ref30] Huisjes R. Satchwell T. J. Verhagen L. P. Schiffelers R. M. van Solinge W. W. Toye A. M.. (2018). Quantitative measurement of red cell surface protein expression reveals new biomarkers for hereditary spherocytosis. Int. J. Lab. Hematol. 40, e74–e77. 10.1111/ijlh.12841, PMID: 29746727

[ref31] Jin J. Y. Tooze J. A. Marsh J. C. W. Gordon-Smith E. C. (1997). Glycosylphosphatidyl-inositol (GPI)-linked protein deficiency on the platelets of patients with aplastic anaemia and paroxysmal nocturnal haemoglobinuria: two distinct patterns correlating with expression on neutrophils. Br. J. Haematol. 96, 493–496. 10.1046/j.1365-2141.1997.d01-2047.x, PMID: 9054654

[ref32] Kahn M. Maley J. Lasker G. Kadowitz P. (2013). Updated role of nitric oxide in disorders of erythrocyte function. Cardiovasc. Hematol. Disord. Drug Targets 13, 83–87. 10.2174/1871529X11313010009, PMID: 23534951 PMC3635483

[ref33] Karnchanaphanurach P. Mirchev R. Ghiran I. Asara J. M. Papahadjopoulos-Sternberg B. Nicholson-Weller A.. (2009). C3b deposition on human erythrocytes induces the formation of a membrane skeleton-linked protein complex. J. Clin. Invest. 119, 788–801. 10.1172/JCI36088, PMID: 19258706 PMC2662546

[ref34] Klei T. R. L. Meinderts S. M. van den Berg T. K. van Bruggen R. (2017). From the cradle to the grave: the role of macrophages in erythropoiesis and erythrophagocytosis. Front. Immunol. 8. 10.3389/fimmu.2017.00073, PMID: 28210260 PMC5288342

[ref35] Kulasekararaj A. G. Hill A. Rottinghaus S. T. Langemeijer S. Wells R. Gonzalez-Fernandez F. A.. (2019). Ravulizumab (ALXN1210) vs eculizumab in C5-inhibitor-experienced adult patients with PNH: the 302 study. Blood 133, 540–549. 10.1182/blood-2018-09-876805, PMID: 30510079 PMC6368201

[ref36] Lin Z. Schmidt C. Q. Koutsogiannaki S. Ricci P. Risitano A. M. Lambris J. D.. (2015). Complement C3dg-mediated erythrophagocytosis: implications for paroxysmal nocturnal hemoglobinuria. Blood 126, 891–894. 10.1182/blood-2015-02-625871, PMID: 26082452 PMC4536542

[ref37] Lutz H. U. (2004). Innate immune and non-immune mediators of erythrocyte clearance. Cell. Mol. Biol. (Noisy-le-Grand) 50, 107–116. 15095782

[ref38] Lutz H. U. Bogdanova A. (2013). Mechanisms tagging senescent red blood cells for clearance in healthy humans. Front. Physiol. 4:387. 10.3389/fphys.2013.00387, PMID: 24399969 PMC3872327

[ref39] Malato A. Saccullo G. Lo Coco L. Mancuso S. Santoro M. Martino S.. (2012). Thrombotic complications in paroxysmal nocturnal haemoglobinuria: a literature review. Blood Transfus. 10, 428–435. 10.2450/2012.0161-11, PMID: 22790262 PMC3496228

[ref40] Ninomiya H. Kawashima Y. Hasegawa Y. Nagasawa T. (1999). Complement-induced procoagulant alteration of red blood cell membranes with microvesicle formation in paroxysmal nocturnal haemoglobinuria (PNH): implication for thrombogenesis in PNH. Br. J. Haematol. 106, 224–231. 10.1046/j.1365-2141.1999.01483.x, PMID: 10444191

[ref500] Peacock-Young B. Macrae F. L. Newton D. J. Ariëns R. A. S. (2018). The prothrombotic state in paroxysmal nocturnal hemoglobinuria: a multifaceted source. Haematologica 103, 9–17. 10.3324/haematol.2017.177618, PMID: 29246924

[ref41] Pollet H. Conrard L. Cloos A.-S. Tyteca D. (2018). Plasma membrane lipid domains as platforms for vesicle biogenesis and shedding? Biomol. Ther. 8:94. 10.3390/biom8030094, PMID: 30223513 PMC6164003

[ref42] Rank A. Nieuwland R. Delker R. Köhler A. Toth B. Pihusch V. (2010). Cellular origin of platelet-derived microparticles *in vivo*. Thromb. Res. 126, e255–e259. 10.1016/j.thromres.2010.07.012 20696467

[ref43] Rapido F. (2017). The potential adverse effects of haemolysis. Blood Transfus. 15, 218–221. 10.2450/2017.0311-16, PMID: 28518048 PMC5448827

[ref44] Risitano A. M. (2012). Paroxysmal nocturnal hemoglobinuria and other complement-mediated hematological disorders. Immunobiology 217, 1080–1087. 10.1016/j.imbio.2012.07.014, PMID: 22964233

[ref45] Risitano A. M. Notaro R. Marando L. Serio B. Ranaldi D. Seneca E.. (2009). Complement fraction 3 binding on erythrocytes as additional mechanism of disease in paroxysmal nocturnal hemoglobinuria patients treated by eculizumab. Blood 113, 4094–4100. 10.1182/blood-2008-11-189944, PMID: 19179465

[ref46] Risitano A. M. Rotoli B. (2008). Paroxysmal nocturnal hemoglobinuria: pathophysiology, natural history and treatment options in the era of biological agents. Biologics 2, 205–222. PMID: 19707355 10.2147/btt.s1420 PMC2721357

[ref47] Saha S. Anilkumar A. A. Mayor S. (2016). GPI-anchored protein organization and dynamics at the cell surface. J. Lipid Res. 57, 159–175. 10.1194/jlr.R062885, PMID: 26394904 PMC4727430

[ref49] Said A. S. Rogers S. C. Doctor A. (2018). Physiologic impact of circulating RBC microparticles upon blood-vascular interactions. Front. Physiol. 8, 1–14. 10.3389/fphys.2017.01120 PMC5770796 29379445

[ref50] Salzer U. Prohaska R. (2001). Stomatin, flotillin-1, and flotillin-2 are major integral proteins of erythrocyte lipid rafts. Blood 97, 1141–1143. 10.1182/blood.V97.4.1141 11159550

[ref51] Salzer U. Zhu R. Luten M. Isobe H. Pastushenko V. Perkmann T. (2008). Vesicles generated during storage of red cells are rich in the lipid raft marker stomatin. Transfusion 48, 451–462. 10.1111/j.1537-2995.2007.01549.x 18067507

[ref52] Sato S. Kozuma Y. Hasegawa Y. Kojima H. Chiba S. Ninomiya H. (2010). Enhanced expression of CD71, transferrin receptor, on immature reticulocytes in patients with paroxysmal nocturnal hemoglobinuria. Int. J. Lab. Hematol. 32, e137–e143. 10.1111/j.1751-553X.2009.01148.x 19302232

[ref53] Sloand E. M. Mainwaring L. Keyvanfar K. Chen J. Maciejewski J. Klein H. G.. (2004). Transfer of glycosylphosphatidylinositol-anchored proteins to deficient cells after erythrocyte transfusion in paroxysmal nocturnal hemoglobinuria. Blood 104, 3782–3788. 10.1182/blood-2004-02-0645, PMID: 15304386

[ref54] Sutherland D. R. Illingworth A. Keeney M. Richards S. J. (2015). “High-sensitivity detection of PNH red blood cells, red cell precursors, and white blood cells” in Current protocols in cytometry (Hoboken, NJ, USA: John Wiley & Sons, Inc.), 6.37.1–6.37.29. 10.1002/0471142956.cy0637s72 25827482

[ref55] Takeda J. Miyata T. Kawagoe K. Iida Y. Endo Y. Fujita T. (1993). Deficiency of the GPI anchor caused by a somatic mutation of the PIG-A gene in paroxysmal nocturnal hemoglobinuria. Cell 73, 703–711. 10.1016/0092-8674(93)90250-T 8500164

[ref56] Whitlow M. Iida K. Marshall P. Silber R. Nussenzweig V. (1993). Cells lacking glycan phosphatidylinositol-linked proteins have impaired ability to vesiculate. Blood 81, 510–516. PMID: 7678519

[ref57] Wiedmer T. Hall S. E. Ortel T. L. Kane W. H. Rosse W. F. Sims P. J. (1993). Complement-induced vesiculation and exposure of membrane prothrombinase sites in platelets of paroxysmal nocturnal hemoglobinuria. Blood 82, 1192–1196. 7688991

[ref58] Willekens F. L. A. Werre J. M. Groenen-Döpp Y. A. M. Roerdinkholder-Stoelwinder B. De Pauw B. Bosman G. J. C. G. M. (2008). Erythrocyte vesiculation: a self-protective mechanism? Br. J. Haematol. 141, 549–556. 10.1111/j.1365-2141.2008.07055.x 18419623

[ref59] Willekens F. L. A. Werre J. M. Kruijt J. K. Roerdinkholder-Stoelwinder B. Groenen-Döpp Y. A. M. Van Den Bos A. G.. (2005). Liver Kupffer cells rapidly remove red blood cell-derived vesicles from the circulation by scavenger receptors. Blood 105, 2141–2145. 10.1182/blood-2004-04-1578, PMID: 15550489

[ref60] Zolla L. D’Alessandro A. (2012). Shaking hands with the future through omics application in transfusion medicine and clinical biochemistry. Blood Transfus. 10, 10–12. 10.2450/2012.001S PMC3418614 22890259

