# Supplementary figures and images for: Red Blood Cell Homeostasis and Altered Vesicle Formation in Patients With Paroxysmal Nocturnal Hemoglobinuria
Source: Front Physiol. 2019 May 15;10:578. doi: 10.3389/fphys.2019.00578 (PMC6529780; doi:10.3389/fphys.2019.00578)

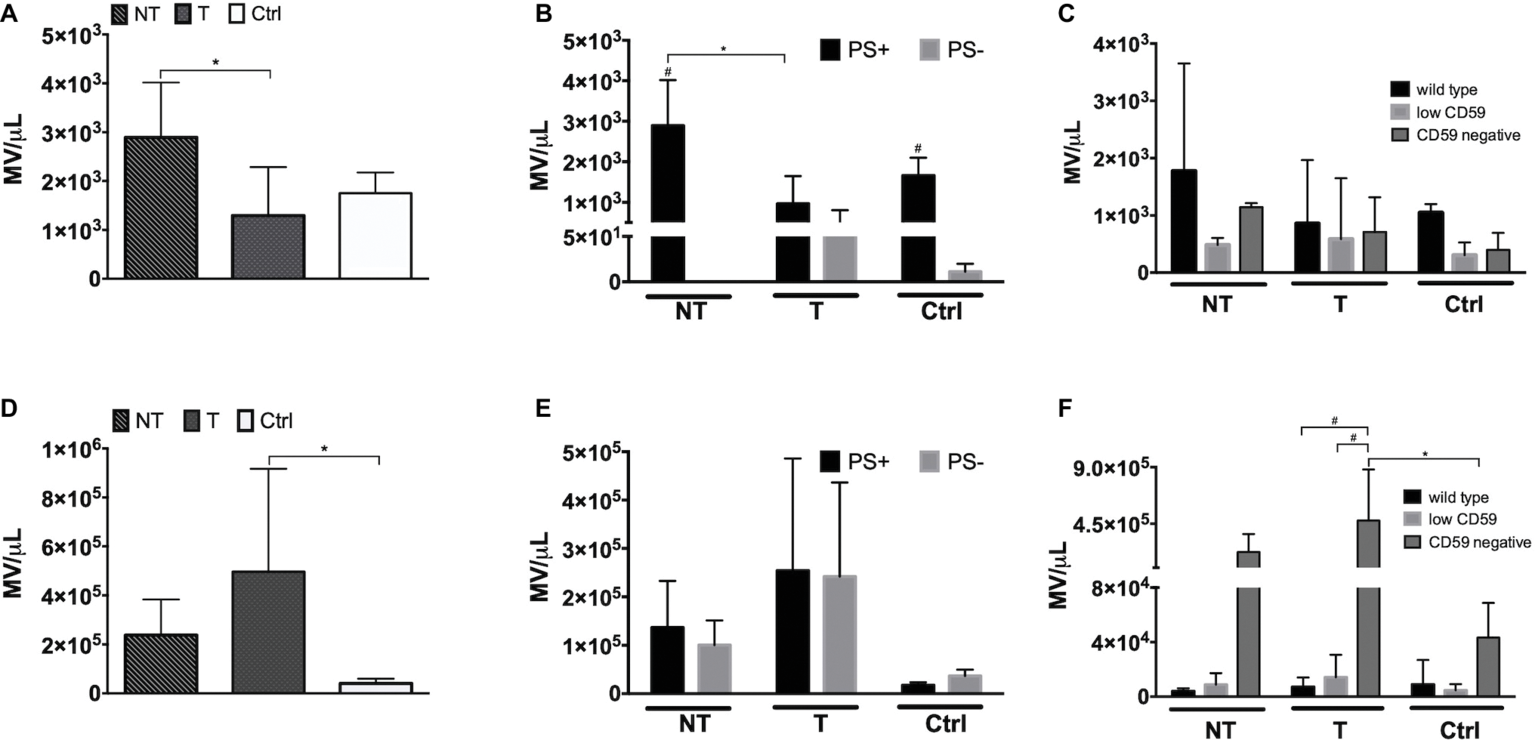

Supplement: SUPPLEMENTARY FIGURE S1 — Microvesicle numbers and composition in the blood of patients with PNH CD59 level and treatment. (A) Concentration (MV/μl) of CD235a-positive, RBC-derived microvesicles (MVs) in the blood of PNH patients who did not receive treatment (NT; N = 3), PNH patients who were treated with eculizumab (T; N = 6), and control healthy donors (N = 6); (B) concentration of RBC-derived microvesicles in the blood of PNH patients who did not receive treatment (NT; N = 3), PNH patients who were being treated with eculizumab (T; N = 6) and control donors (N = 6) according their reactivity to Annexin V (phosphatidylserine positive (PS+) or negative (PS−); (C) RBC-derived microvesicles in the blood of PNH patients who did not receive treatment (NT; N = 3), PNH patients who were being treated with eculizumab (T; N = 6) categorized in wild type, CD59-low and CD59-negative as described for RBCs (Materials and Methods); (D) concentration of platelet-derived microvesicles (CD41-positive) from the blood of PNH patients who did not receive treatment with eculizumab (NT; N = 3), PNH patients who were being treated with eculizumab (T; N = 6) and control healthy donors (N = 6); (E) concentration of platelet-derived microvesicles in the blood of PNH patients who did not receive treatment with eculizumab (NT; N = 3), PNH patients who were being treated with eculizumab (T; N = 6) and control healthy donors (N = 6), according their reactivity to Annexin V (PS+ or PS−); (F) platelet-derived microvesicles from the blood of PNH patients who did not receive treatment with eculizumab (NT; N = 3), PNH patients who were being treated with eculizumab (T; N = 6) were categorized in wild type, low CD59, and CD59-negative as described for RBCs and quantified and analyzed by flow cytometry as described before (see Materials and Methods). #Significantly different from the other parameter in the same group (p < 0.05). *Significantly different between groups (p < 0.05). [file Image_1.tif]

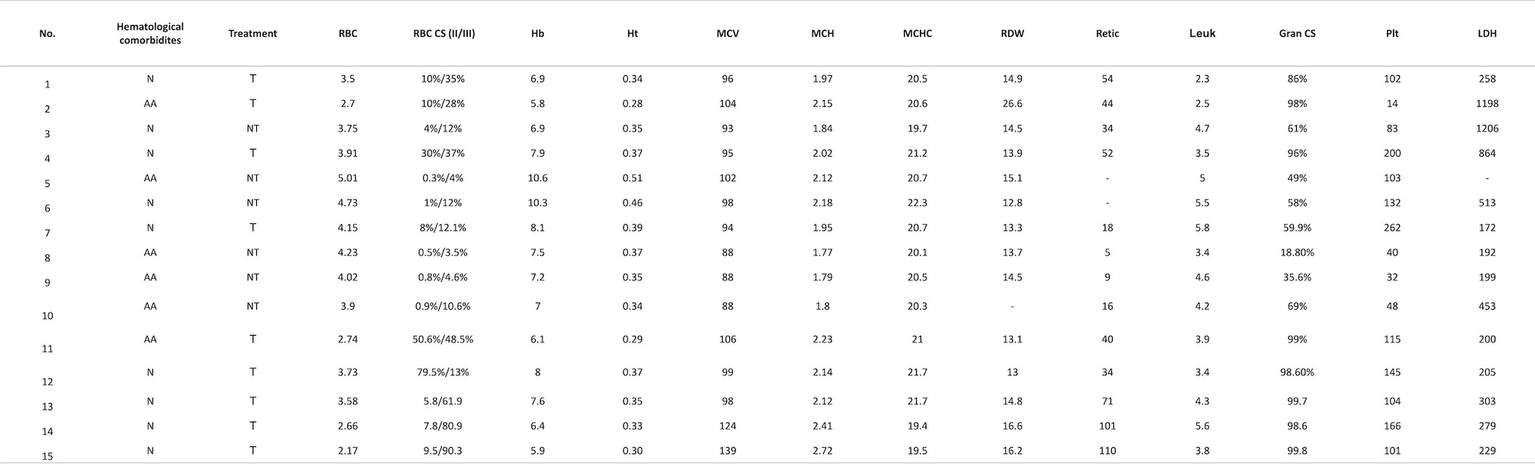

Supplement: SUPPLEMENTARY TABLE 1 — Clinical data of PNH patients. AA, PNH patient with aplastic anemia; N, PNH patient without hematological comorbidities; T, in treatment; NT, not in treatment; RBC, red blood cells (×1012/L); RBC CS (II/III), red blood cell clone size (type II and III); Hb, hemoglobin (g/dl); Ht, hematocrit (%); MCV, mean corpuscular volume (fl); MCH, mean corpuscular hemoglobin (pg); MCHC, mean corpuscular hemoglobin concentration (g/dl); RDW, red blood cell distribution width (%); Retic, reticulocytes (promille); RBC Tr, red blood cell transfusion in the last 3 months; Leuk, Leukocytes (×109/L); Gran CS., granulocytes clone size; Plt, platelet (×109/L); LDH, lactate dehydrogenase (U/L); −, not available. Reference values for healthy adults: RBC: for men, 4.7–6.1 × 10^12^/L and for women, 4.2–5.4 × 10^12^/L; Hb: for men, 8.5–11 mmol/L and for women, 7.5–10 mmol/dl; Ht: for men, 0.4–0.54 and for women, 0.36–0.46; MCV: 80–96 fl; MCH: 1.7–2.1 fmol; MCHC: 19.3–22.5 mmol/L; RDW: 11.5–14.5%; Retic: 8–26 promille; Leuk: 4.5–11 × 109/L; Plt: 150–400 × 109/L; LDH: 135–225 U/L. [file Image_2.tif]
